# Mycorrhiza Reduces Adverse Effects of Dark Septate Endophytes (DSE) on Growth of Conifers

**DOI:** 10.1371/journal.pone.0042865

**Published:** 2012-08-10

**Authors:** Vanessa Reininger, Thomas N. Sieber

**Affiliations:** Department of Environmental Systems Science, ETH Zurich, Zurich, Switzerland; Nanjing Agricultural University, China

## Abstract

Mycorrhizal roots are frequently colonized by fungi of the *Phialocephala fortinii* s.l. – *Acephala applanata* species complex (PAC). These ascomycetes are common and widespread colonizers of tree roots. Some PAC strains reduce growth increments of their hosts but are beneficial in protecting roots against pathogens. Nothing is known about the effects of PAC on mycorrhizal fungi and the PAC-mycorrhiza association on plant growth, even though these two fungal groups occur closely together in natural habitats. We expect reduced colonization rates and reduced negative effects of PAC on host plants if roots are co-colonized by an ectomycorrhizal fungus (ECM). Depending on the temperature regime interactions among the partners in this tripartite ECM-PAC-plant system might also change. To test our hypotheses, effects of four PAC genotypes (two pathogenic and two non-pathogenic on the Norway spruce), mycorrhization by *Laccaria bicolor* (strain S238N) and two temperature regimes (19**°**C and 25**°**C) on the biomass of the Douglas-fir (*Pseudotsuga menziesii*) and Norway spruce (*Picea abies*) seedlings were studied. Mycorrhization compensated the adverse effects of PAC on the growth of the Norway spruce at both temperatures. The growth of the Douglas-fir was not influenced either by PAC or mycorrhization at 19°C, but at 25°C mycorrhization had a similar protective effect as in the Norway spruce. The compensatory effects probably rely on the reduction of the PAC-colonization density by mycorrhizae. Temperature and the PAC strain only had a differential effect on the biomass of the Norway spruce but not on the Douglas-fir. Higher temperature reduced mycorrhization of both hosts. We conclude that ectomycorrhizae form physical and/or physiological barriers against PAC leading to reduced PAC-colonization of the roots. Additionally, our results indicate that global warming could cause a general decrease of mycorrhization making primary roots more accessible to other symbionts and pathogens.

## Introduction

Biocenoses are composed of communities of different organisms which share the same habitat but presumably have their own niches. Interactions among the inhabitants living spatially closely together [1]–[3] can have different characteristics and span the whole range from mutualism to parasitism [4]–[7]. Some of these interactions are known to be costly at least for one of the partners, whereas others are beneficial for both partners. Arbuscular mycorrhizal (AM) and ectomycorrhizal (ECM) fungi live in symbiosis with their host plants, and host and fungal partner mutually profit [7] from this interaction [8]. Dark septate endophytes (DSE) are also common root colonizers of mycorrhizal plants [9]–[11], even though their interaction is not as clearly beneficial as that between plants and mycorrhizal fungi [12]–[14]. However, DSE are frequently isolated from mycorrhizal root tips [15]–[17], and therefore, share their habitat with mycorrhizal fungi [18]. Consequently, interactions between mycorrhizal fungi and DSE are inevitable and may have beneficial, adverse or no effects on the host plant.

Ascomycetous fungi of the *Phialocephala fortinii* s.l. – *Acephala applanata* species complex (PAC) are the main component of DSE fungi, and they are very common on several woody plant species throughout the Northern hemisphere [9], [10], [17], [19]–[25]. Reports about the effects of PAC on plant performance are contradictory [17]. In a recent study about PAC-Norway spruce interactions most of the more than 30 genotypically different PAC strains had little effects, but a few strains were highly virulent [13]. Interestingly, none of the strains stimulated plant growth.

Interactions of plants with endophytes or mycorrhizal fungi were studied intensively [13], [26]–[30], even under different temperature regimes [31]. However, studies about tripartite interactions among endophytes, mycorrhizal fungi and plants are scarce [32]–[35], prompting Porras-Alfaro and Bayman [36] to emphasize the need for integrating mycorrhizal and fungal root-endophyte research. Studies about interactions among PAC, ectomycorrhizal fungi and plants are missing completely. We hypothesize that ectomycorrhizae (ECM) impede colonization of primary roots by PAC, hereby reducing negative effects of virulent PAC strains on plant growth. ECM may inhibit PAC either directly or indirectly. Direct inhibition may occur by metabolic (e.g. production of antibiotic metabolites) or mechanical defense (e.g. mycelial mantles constituting physical barriers [37]), indirect inhibition by strengthening plant defense or by competition for nutrients. Additionally, we suppose that these interaction patterns change if environmental mean temperature changes. For example, if the climate warms up, Douglas-fir (*Pseudotsuga menziesii*) might replace Norway spruce (*Picea abies*) at the dispersal limits where high temperature constrains regeneration of Norway spruce since introduced Douglas-fir is better adapted to higher temperatures and is meanwhile well established in European forests.

**Table 1:** PAC strains included in this study.

ETH -strainnumber	Strainlabel	Species	Pathogenicity^a^	Original host	Allele length of locus mPF_142B [bp]
6_2_7v	A	*Phialocephala subalpina sssubalpinainasubalpina*	+	*Vaccinium myrtillus myrtmyrtillus*	174
6_37_6v	B	*Phialocephala subalpina*	–	*Vaccinium myrtillus*	162
7_45_5	C	*Phialocephala fortinii* s.s.	–	*Picea abies*	154
7_63_4	D	*Phialocephala fortinii* s.s.	+	*Picea abies*	152

a Pathogenicity on Norway spruce seedlings according to Tellenbach *et al.* [13].

To test PAC-ECM-host interactions, an experiment was set up with the ectomycorrhizal model strain *Laccaria bicolor* S238N [38]–[40], four PAC strains differing in virulence against Norway spruce and Douglas-fir as hosts and two temperature regimes. *L. bicolor* was chosen as ECM because of its strong competitive behavior on Douglas-fir [41] and due to its distinctness from PAC mycelium in regards to color and presence of clamp connections. Douglas-fir and Norway spruce were chosen as host plants since they are clearly hosts of PAC, and PAC is known to behave completely different on different hosts [30]. Additionally Norway spruce could be replaced by Douglas-fir under global warming. The experiment was conducted under two temperature regimes which correspond to actual and predicted temperature conditions on mountain slopes in summer with southern aspect [42] to account for a global warming scenario.

**Table 2:** Factors and their two-fold interactions which are retained in the reduced model [1] for response variable ‘plant biomass’ including both hosts.

Source of Variation	Df	Mean Sq	p-values
PAC strain	3	0.04129	0.0003597*
Host	1	1.46641	<0.0001*
Temperature	1	0.80143	<0.0001*
Mycorrhization	1	0.80393	<0.0001*
Block	1	0.01729	0.1035443
PAC strain:Temperature	3	0.01496	0.077052
PAC strain:Mycorrhization	3	0.01403	0.0925703
Host:Temperature	1	0.81837	<0.0001*
Host:Mycorrhization	1	0.15225	2.27E-06*
Residuals	232	0.00647	

* = significant at α = 0.05.

## Material and Methods

### Fungal Strains, Culture Conditions and Inoculation

Two strains each of two PAC species [30] differing in virulence on Norway spruce [13] (Table 1) and *Laccaria bicolor* strain S238N (isolated 1976 from *Tsuga mertensiana* in Crater Lake National Park, Oregon, USA) as an ectomycorrhizal model strain [39] were grown for five weeks in 100 ml Erlenmeyer flasks containing 50 ml of either 20 g l^−1^ malt extract for PAC or liquid Pachlewski medium (concentration per liter: 7.3 mM KH_2_PO_4_, 5 mM (D+)-Glucose, 2.7 mM C_4_H_12_N_2_O_6_, 7.3 mM MgSO_4_ heptahydrat, 2.9 mM thiamine-HCL, 1 ml trace-element stock solution) for *L. bicolor* on a shaker at 20°C and 85 rpm. For inoculation of PAC and *L. bicolor*, mycelium was washed with sterile high-purity water (Barnstead NANOpure DIamond™, Skan AG, Allschwil, Switzerland) under sterile conditions. The concentration of mycelial inocula was adjusted to 0.015 g ml^−1^ fresh mycelium with sterile high-purity water and 2 ml of this suspension were used per experimental unit. Tubes of 100 ml filled with sterile 1∶100 peat : vermiculite (v:v) substrate (pH 5.0) - soaked with liquid Pachlewski medium - served as experimental units. This substrate allowed growth of *L. bicolor* in contrast to 1∶1 peat : vermiculite (v:v) used in previous experiments [13], [30]. Tubes assigned to ‘mycorrhization’ treatments were inoculated with *L. bicolor* and incubated for 5½ weeks at 20°C prior to planting the tree seedlings. Seeds of *Picea abies* (Birmenstorf Tannwald, Aargau, Switzerland, 400 m NN, year 1987) and *Pseudotsuga menziesii* var. *menziesii* (Biel Vorberg, Bern, Switzerland, 620–730 m NN, year 2009) were surface-sterilized for 30 and 90 minutes with 30% H_2_O_2_, respectively, rinsed in EtOH for 10 s, and germinated on H_2_O agar. A sterile seedling of either Norway spruce or Douglas-fir was planted per tube and incubated in a phytotron (see below). After three weeks PAC inoculum was added to the seedlings assigned to the ‘PAC’ treatments (see above).

**Figure 1:**
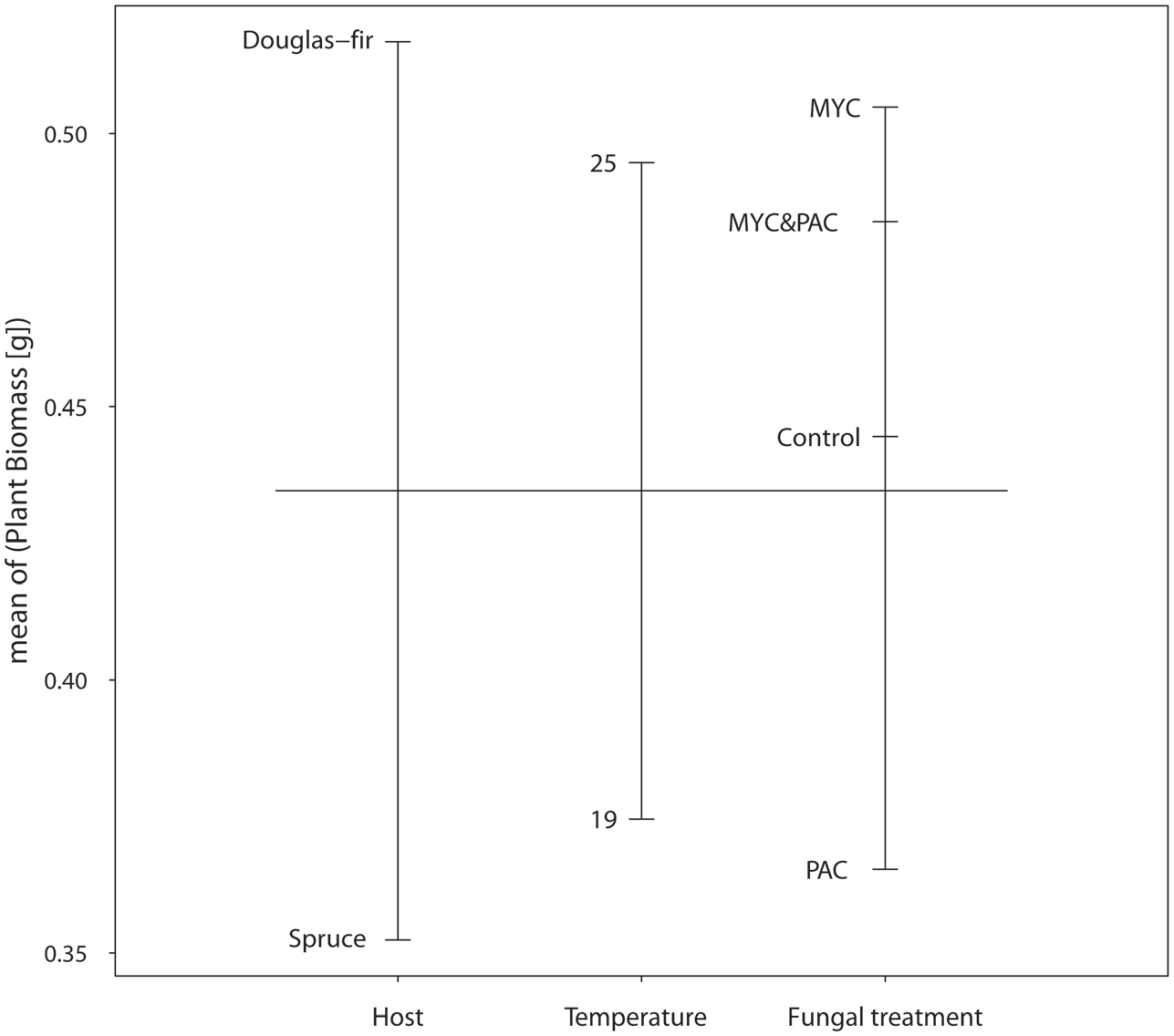
Effects of ‘host’, ‘temperature’ and ‘fungal treatment’ on mean plant dry weight [g]. MYC  =  plants with mycorrhiza formed by *Laccaria bicolor* S238N; PAC  =  plants colonized by either one of the PAC strains (see Table 1); MYC&PAC  =  plants with both mycorrhiza and PAC colonization; Control  =  plants without any fungal treatment. Design plot drawn according to Crawley [60].

### Experimental Setup

The experiment was setup as a completely randomized block design with two complete blocks, each containing all treatments, to account for the environmental heterogeneity, though small, in the growth chamber [16 h day (120–140 µEm^−2^s^−1^)/8 h night (including a 2 h ramp at the start and the end of the day during which temperature and light increased and dropped slowly), temperature (22°C/15°C), and 45% relative humidity (RH)] and run for five months after PAC inoculation. Two temperatures were applied by immersion of the lower ¾ of the tubes in water baths. The average daily temperature in the tubes was controlled using dataloggers (iButtons® Maxim Integrated Products, Inc., CA, USA) and set to 19°C and 25°C, respectively. Each of the four PAC strains A, B, C, and D (see Table 1) was added separately to tubes containing *L. bicolor*, and each PAC strain and *L. bicolor* were inoculated singularly. Fungus-free tubes served as negative controls. The combination of all possible factor levels resulted in 40 treatments [5 levels of PAC (without PAC, strains A, B, C, and D)×2 levels of mycorrhization (with and without *L. bicolor*)×2 temperature treatments×2 host species]. Eight tubes were prepared as replicates per treatment. Plants were watered three times a week with deionized water as needed. Depending on the experimental stage, each tube received 3–7 ml of a 1 ml l^−1^ WUXAL solution (Universaldünger, Maag, Syngenta Agro AG, Dielsdorf, Switzerland) as fertilizer once a month.

**Figure 2:**
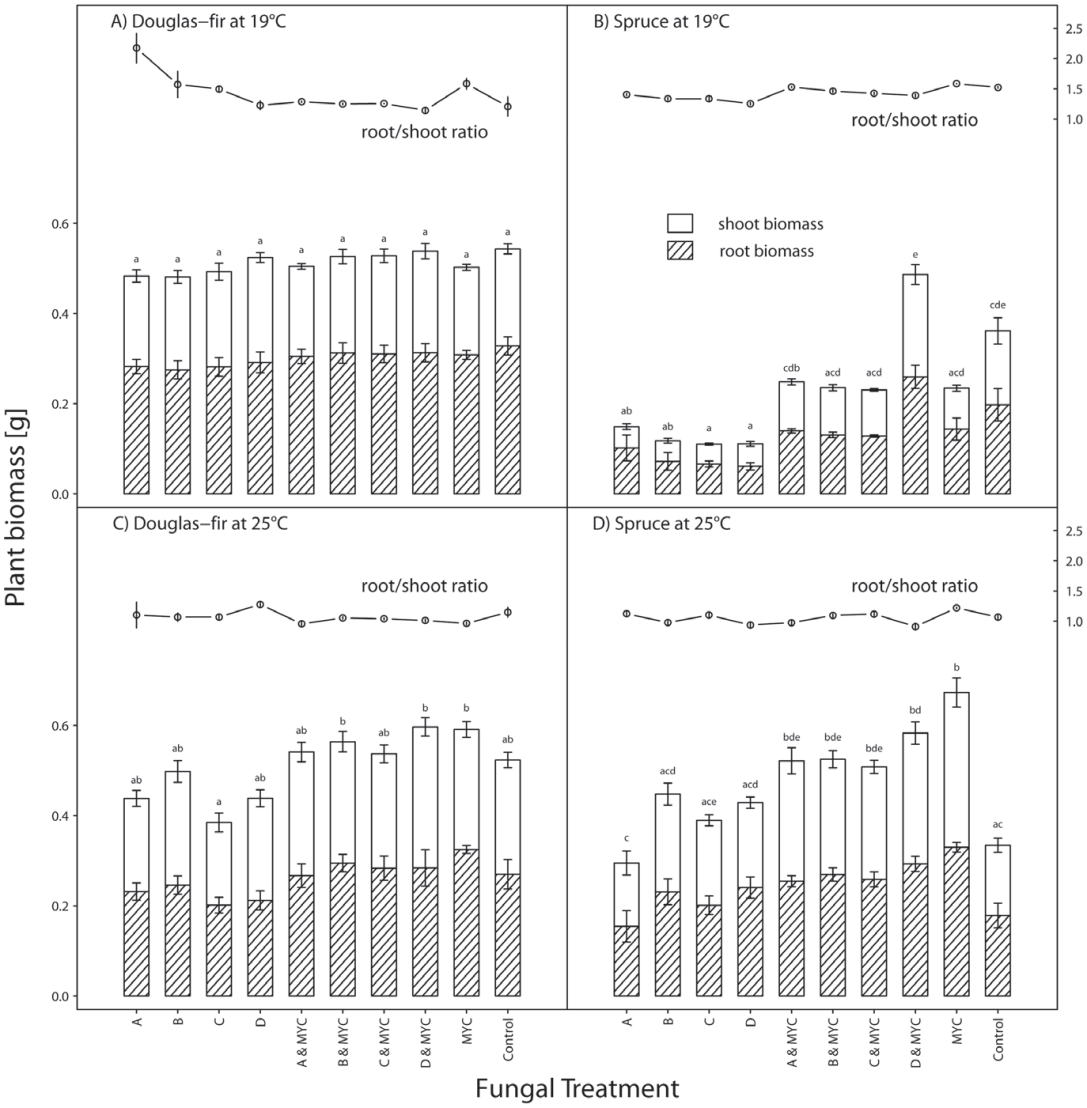
The effects of ‘host’, ‘temperature’ and ‘fungal treatment’ on host dry weight and the root/shoot ratio. Total host biomass is displayed as the sum of root (hatched) and shoot (empty) biomass. MYC indicates ‘mycorrhization’ by *Laccaria bicolor* S238N and letters A, B, C, and D inoculation with PAC strains A, B, C, or D (see Table 1). Panel (A) shows the data for Douglas-fir at 19°C, (B) for Norway spruce at 19°C, (C) for Douglas-fir at 25°C and (D) for Norway spruce at 25°C. Error bars show the standard error of the mean for root and shoot biomass. The root/shoot ratio is displayed as the mean for each fungal treatment and the control including the standard error of the mean. Different letters above the bars indicate significant differences in total biomass at α = 0.05 between treatments according to Tukey’s HSD.

### Sampling and Data Collection

Roots from all eight replicates of each treatment were rinsed carefully under running tap water to remove the peat-vermiculite. The degree of mycorrhization of the whole root system was scored under a binocular in large glass Petri dishes filled with insipid tap water using the following classification system: 0 =  no mycorrhization, 1 = 1–25% of the root tips mycorrhized, 2 = 26–50%, 3 = 51–75%, and 4 = 76–100%.

**Table 3:** Factors and their interactions which are retained in the reduced model [1] for response variable ‘plant biomass of Douglas-fir’.

Source of Variation	Df	Mean Sq	p-values
Temperature	1	0.001304	0.6872
Mycorrhization	1	0.215359	<0.0001*
Block	1	0.012354	0.2166
Temperature : Mycorrhization	1	0.10695	0.0004*
Temperature : Block	1	0.019461	0.1217
Residuals	114	0.008003	

* = significant at α = 0.05.

Root pieces of 0.5 cm length were excised for DNA extraction and reisolation to detect and quantify PAC of three replicates per treatment. The root segments were excised as follows. Three main roots were selected per plant, and on every root seven segments were randomly cut for DNA extraction and one for reisolation. The 21 segments from the three roots for DNA extraction were pooled, freeze dried and weighed. To estimate biomass of each PAC strain, 3 mg of freeze-dried reference mycelium was added before DNA extraction (mycelium of strain C was added as reference to root samples containing strain A, mycelium of strain A to root samples containing strains B, C and D; for details see Reininger *et al.* [43]) and stored at −80°C until further processing (see ‘root-reference-mixtures’ below). The three fresh root segments for PAC reisolation were surface-sterilized in 30% H_2_O_2_ for 30 s and 10 s in EtOH and incubated on terramycin-malt agar (20 g l^−1^ malt extract, 15 g l^−1^ agar, 50 mg l^−1^ terramycin®) at room temperature. The remainder of the seedlings were cut into roots and shoot, dried at 50°C for 48 hours and weighed.

**Table 4:** Factors and their interactions which are retained in the reduced model [1] for response variable ‘plant biomass of Norway spruce’.

Source of Variation	Df	Mean Sq	p-values
Temperature	1	2.02066	<0.0001*
PAC strain	3	0.06633	<0.0001*
Mycorrhization	1	0.84077	<0.0001*
Block	1	0.02822	0.0597
Temperature : Mycorrhization	1	0.00878	0.2907
Temperature : PAC strain	3	0.02123	0.0477*
PAC strain : Mycorrhization	3	0.04432	0.0012*
Temperature : PAC strain : Mycorrhization	3	0.04298	0.0014*
Residuals	111	0.00779	

* = significant at α = 0.05.

### DNA Extraction, Microsatellite PCR and Microsatellite Fragment Analysis

Frozen root-reference-mixtures were homogenized in 2 ml safe-lock tubes, using a Retsch machine MM 200, adding a small metal ball and a few grains of sand. DNA extraction followed the manufacturers protocol of the DNeasy plant mini kit (Qiagen, Hilden, Germany) except for the lysis buffer which was replaced by hexadecyltrimethylammonium bromide (CTAB) according to Rogers *et al.* [44] and Rogers and Bendich [45]. Microsatellite PCR was performed in 15 µl volumes containing 2 µl 1∶50 diluted DNA, 50 mM KCl, 10 mM Tris-HCl, 1,5 mM MgCl_2_, 200 µM dNTPs (Amersham Pharmacia Biotech), 0,4 µM forward and reverse primer (F: GCTTTCACATCACCATCCAG; R: GGTGAGTTGGTTGCGAGTTT) and 0,3 U Taq polymerase (Amersham Pharmacia Biotech). The running conditions were 2 min at 94°C followed by 33 cycles of denaturation for 30 s at 94°C, annealing for 30 s at 53°C and extension for 30 s at 72°C (followed by a final extension step of 10 min at 72°C) [46]. For the microsatellite fragment analysis 15–fold diluted amplicons of the PCRs were prepared and 4 µL of the dilutions were mixed with 9.05 µL Hi-Di™ formamide and 0.25 µL GeneScan™ 500 LIZ™ (Applied Biosystems). Fragment lengths and the area under the light emission curve (AUC) of each fragment were measured using an ABI 3730×l DNA Analyzer (Applied Biosystems) and analyzed using the GeneMapper v. 4.0 software (Applied Biosystems) [47]. Biomass of PAC mycelia in and on roots was estimated using the method described in Reininger *et al*. [43].

**Figure 3:**
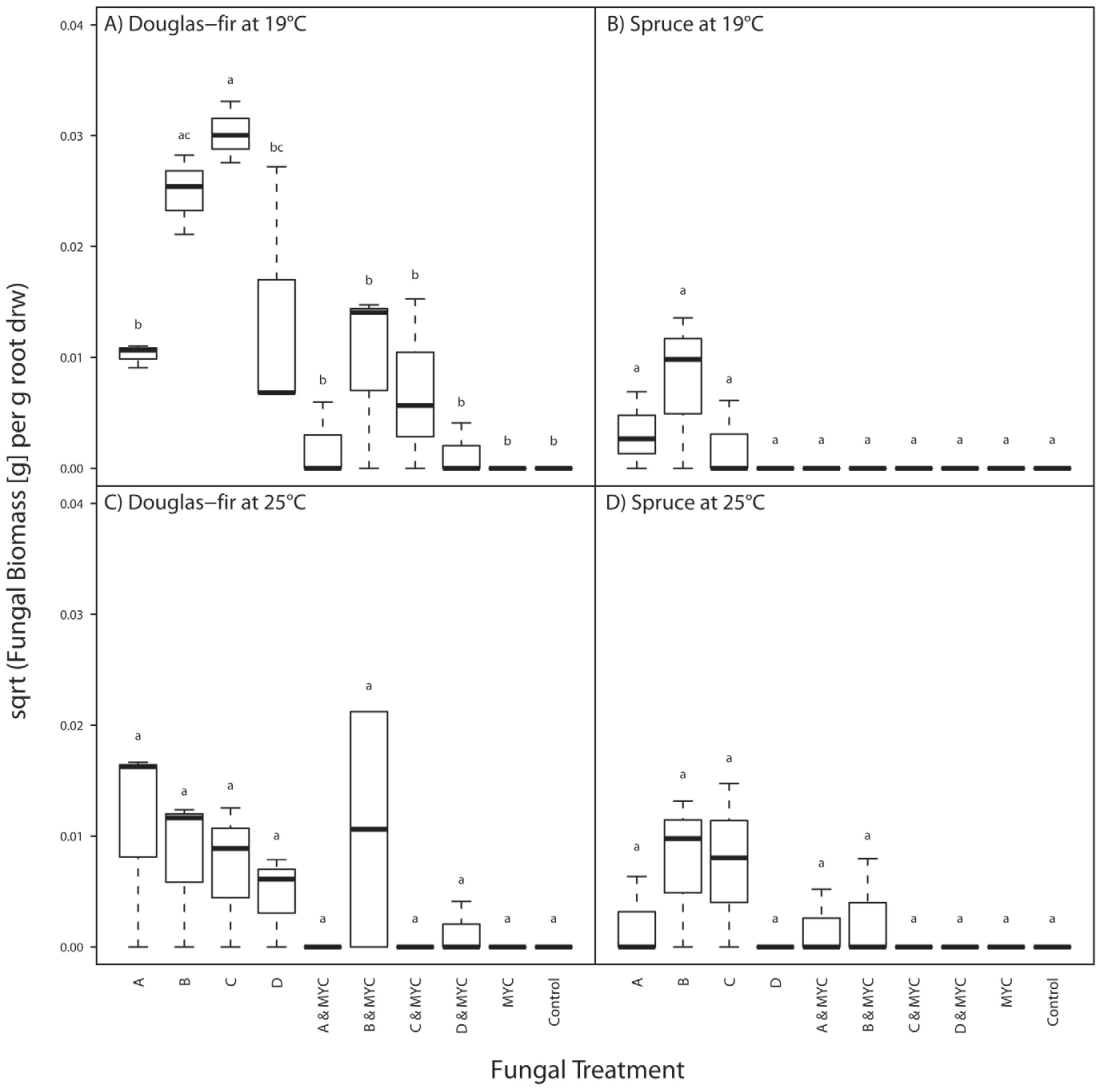
Boxplots showing PAC biomass [g] depending on the ‘fungal treatment’, the ‘host species’ and ‘temperature’. MYC indicates ‘mycorrhization’ by *Laccaria bicolor* S238N and letters A, B, C, and D inoculation with PAC strains A, B, C, or D (see Table 1). The plots are drawn according to Crawley [60]: The bold horizontal line shows the median. The bottom and top of the box show the 25th and 75th percentiles, respectively. The vertical dashed lines (‘whiskers’) show either the maximum value or 1.5 times the interquartile range of the data, whichever is the smaller. Points more than 1.5 times the interquartile range above or below the third or first quartile are defined as outliers and plotted individually, respectively. Different letters above boxes indicate significant differences between treatments; α = 0.05.

### Statistical Analysis

Nine plants had to be excluded from the plant biomass, root/shoot ratio and mycorrhization degree analyses because mycorrhization failed. Additionally, the ‘fungus-free’ and the ‘mycorrhization only’ controls were not included in the analyses of variance of the plant biomass parameters because the emphasize of this study laid on testing the influence of temperature and the inoculation with the mycorrhizal fungus *L. bicolor* on PAC-treated plants. The following multifactorial models were tested (µ  =  overall mean; all possible interactions among factors were also calculated but are not shown in the models below but see Table S1, Table S2, Table S3, and Table S4):
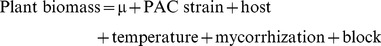
(1)

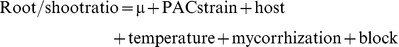
(2)

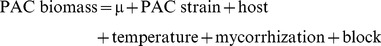
(3)

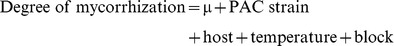
(4)


All models were calculated with and without the factor ‘host’. The factor ‘mycorrhization’ in the models [1]–[3] is binary with 1 =  *L. bicolor* added and 0 =  no *L. bicolor* added (see above). The best transformation was sought comparing residual analyses (Tukey-Anscombe plot, Q-Q plot, leverage plot). Reduced models were calculated and the Akaike information criterion (AIC) was used to find the reduced model that did not significantly reduce the fit of the full model. Tukey’s honest significant differences (TukeyHSD) were calculated for pairwise comparison of effects of factor levels. The software R was used for all statistical analyses [48].

**Figure 4:**
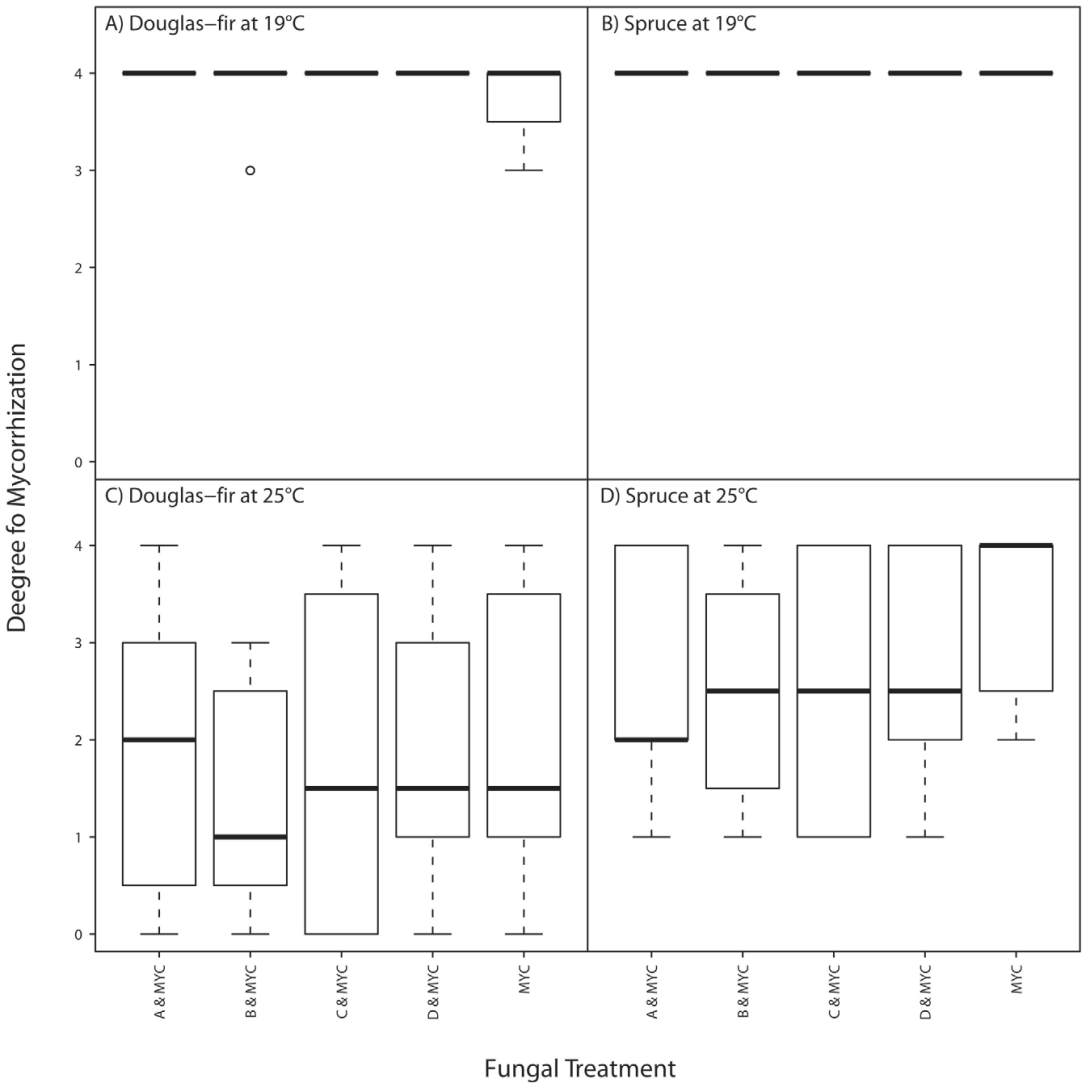
‘Degree of mycorrhization’ by *Laccaria bicolor* S238N depending on ‘host species’, ‘PAC strain’ and ‘temperature’ (degrees of mycorrhization: 1 = 1–25% of the roots mycorrhized, 2 = 26–50%, 3 = 51–75%, 4 = 76–100%). MYC indicates ‘mycorrhization’ by *Laccaria bicolor* S238N and letters A, B, C, and D inoculation with PAC strains A, B, C, or D (see Table 1). There are no significant differences between any two ‘fungal treatments’ within one ‘host’ and ‘temperature’. The plots are drawn according to Crawley [60].

## Results

### Mycorrhization and PAC Detection Using Microsatellites and Reisolations

With the exception of nine plants, mycorrhization occurred in all root systems inoculated with *L. bicolor*. PAC could be detected in 61% of the roots inoculated with PAC using the classical reisolation method but in only 44% using microsatellite analysis, i.e. PAC was detected in a total of 64% of the plants inoculated with PAC using either method.

### Effects of Fungal Treatment and Temperature on Plant Biomass

Root and shoot biomass were highly correlated, and statistical analyses with total plant biomass as response variable led to the same results as the analyses of root and shoot biomass. Consequently, total biomass was used as response variable for further analyses except for the analysis of the root/shoot biomass ratio.

All factors except ‘block’ had a highly significant influence on plant biomass (Table 2 and Table S1) with the factor ‘host’ affecting plant biomass the most (Figure 1). The factor ‘host’ was also responsible for the highly significant interactions between ‘host’, ‘temperature’ and ‘mycorrhization’ (Table 2 and Table S1), indicating that the two hosts reacted differently to ‘temperature’ and ‘mycorrhization’. Therefore and to enhance comprehensibility, data were analyzed separately for each host. This makes also sense from a biological point of view since biomass of the un-inoculated controls of the two hosts differed significantly under the conditions applied in this experiment, indicating different genetic disposition of the two host species.

In general, plants grew better at higher temperature, and if plants were mycorrhized or mycorrhized in combination with PAC compared to fungus-free (controls) or PAC colonized plants (Figure 1). However, as mentioned above, hosts need to be analyzed separately to correctly appreciate the influence of these factors.

### Biomass of Douglas-fir

The fungus-free Douglas-fir controls performed equally well as plants inoculated with any of the PAC strains and/or *L. bicolor* and temperature had no effect (Figure 2A and 2C). Biomass of fungus-free control plants was higher, though not significantly, than biomass of mycorrhized controls at 19°C. At 25°C, it was opposite.

Only ‘mycorrhization’ and the interaction between ‘temperature’×‘mycorrhization’ had a significant effect on the biomass of Douglas-fir whereas ‘temperature’ alone had no significant effect but was retained in the model (Figure 2, Table 3 and Table S1). Mycorrhization had an influence on Douglas-fir biomass only at 25°C. At this temperature, biomass was overall higher in mycorrhized plants (Figure 2C). For example, mycorrhized plants inoculated with PAC strain B or D had significantly higher biomass than non-mycorrhized plants inoculated with strain C. However, considering each PAC strain separately, mycorrhization had no significant influence on plant biomass gain (Figure 2C).

Whereas temperature had no effect on plant biomass, it had a significant effect on the root/shoot ratio since lower temperature promoted root growth and higher temperature shoot growth (Figure 2A and 2C). Mycorrhization had a weak but significant effect on the root/shoot ratio, with mycorrhized plants having a lower ratio, especially at 19°C (Figure 2A and Table S2), i.e. root biomass was lower compared to shoot biomass in plants with mycorrhization. The ‘PAC strain’ had no effect at all and was not even retained in the reduced model.

### Biomass of Norway Spruce

Norway spruce controls performed better at 19°C and worse at 25°C than plants inoculated with PAC and/or *L. bicolor* (Figure 2B and 2D). Biomass of fungus-free control plants was higher, though not significantly, than biomass of mycorrhized controls at 19°C. At 25°C, it was opposite with a significant difference.

‘Temperature’, ‘PAC strain’, ‘mycorrhization’ and some of the interactions were highly significant and therefore retained in the reduced model (Table 4 and Table S1). Biomass of mycorrhized, PAC-colonized Norway spruce was higher (in some cases significantly) than that of non-mycorrhized, PAC-colonized Norway spruce at both temperatures with the difference being more pronounced at 19°C (Figure 2B and 2D). PAC-colonized, mycorrhiza-free Norway spruce performed better at 25°C compared to 19°C. ‘Mycorrhization’ combined with colonization by strain D led to significantly higher biomass at 19°C than any other treatment including the fungus-free controls (Figure 2B). In contrast, D had similar effects as the other PAC strains at 25°C (Figure 2D). The root/shoot ratio was affected only by temperature indicating that roots accumulated more biomass relative to shoots at lower temperature (Figure 2B, 2D and Table S2).

### Effects of Fungal Treatment, Host Species and Temperature on PAC Biomass

Douglas-fir was more densely colonized by PAC than Norway spruce (Figure 3). Colonization by PAC was inhibited by mycorrhization in both host plants at both temperatures (Figure 3).

### Endophytic PAC Biomass in Douglas-fir

‘PAC strain’, ‘temperature’, ‘mycorrhization’ and ‘block’ as well as the interactions between ‘PAC strain’×‘temperature’ and ‘temperature’×‘mycorrhization’ were significant factors in the reduced model (Table S3). Biomass of most PAC strains was higher at 19°C than at 25°C and in mycorrhiza-free plants. At 25°C, fungal treatments had no differential effect on PAC biomass according to Tukey’s HSD whereas at 19°C biomass of strain C was significantly higher than that of all other strains except biomass of strain B in mycorrhiza-free roots (Figure 3A and 3C).

### Endophytic PAC Biomass in Norway Spruce

The full model for Norway spruce could not be reduced using the ‘stepAIC’ procedure even though ‘PAC strain’ and ‘mycorrhization’ were the only significant factors in the model (Table S3). ‘Temperature’ had no significant influence on PAC biomass in Norway spruce seedlings (Figure 3B, 3D and Table S3). Figure 3 and the statistical model suggest that mycorrhization significantly inhibited colonization by PAC, but biomass of none of the PAC strains was significantly reduced by mycorrhization according to pairwise comparisons (Figure 3B and 3D). At 19°C, PAC could only be detected by reisolation in mycorrhized Norway spruce roots, whereas at 25°C PAC strains A and B could be detected in some mycorrhized roots also using microsatellite analysis (Figure 3B and 3D).

### Degree of Mycorrhization

The degree of mycorrhization was significantly influenced by ‘temperature’ and ‘block’ but not by ‘host’ or ‘PAC strain’ (Figure 4 and Table S4). The degree of mycorrhization was higher at 19°C than at 25°C. Since the host had no significant influence on the degree of mycorrhization, calculations for each host separately were not meaningful and hence neglected. Only little and inconsistent variation (between hosts) in the degree of mycorrhization could be observed due to ‘PAC strain’ (Figure 4C and 3D).

## Discussion

This experiment was set up to study a complex network of interactions between dark septate endophytes, mycorrhiza, plants and temperature. Mycorrhization reduced the adverse effects of PAC on growth of Douglas-fir only at 25°C but not at 19°C, i.e. Douglas-fir appears to react indifferently to mycorrhization at 19°C. However, non-mycorrhized, PAC-colonized plants invested more into root compared to shoot growth than mycorrhized, PAC-colonized plants, as indicated by the root/shoot ratios (Figure 2A and Table S2). Probably, this indicates even better root growth of non-mycorrhized, PAC-colonized Douglas-fir compared with mycorrhized, PAC-colonized Douglas-fir. ‘Temperature’, ‘PAC strain’ and ‘mycorrhization’ significantly affected biomass accumulation of Norway spruce and all these factors interacted with each other; even the 3-way interaction was significant, indicating a complex interplay among these three factors. The strain effects were different from those observed by Tellenbach *et al.* [13]. Strain A was the most virulent and reduced plant growth the most in the study of Tellenbach *et al.* [13], whereas it behaved similar as the other strains in this study. The different behaviors are probably due to differences in the potting media and the timing of inoculation. Whereas the potting medium was 1∶1 peat : vermiculite (v:v) in Tellenbach *et al.* [13], we used 1∶100 peat : vermiculite (v:v) to adjust the pH to 5.0. In addition, the medium was completely colonized by PAC mycelium at the moment of planting the seedlings in Tellenbach *et al.* [13], whereas we added PAC to the *L. bicolor* colonized potting medium and after the seedlings had already three weeks to adapt and establish in the pots. Looking at single strains, mycorrhization significantly reduced adverse effects of strain D at 19°C and that of strain A at 25°C, indicating some degree of temperature-dependence of PAC control by mycorrhiza.

Detection of PAC using the classical reisolation technique was more successful than detection using the microsatellite method. This contrasts with Reininger *et al.* [43] who had more success using the microsatellite method. Apart from the different PAC inoculation method used by Reininger *et al.* [43] differences to our results are mainly of stochastic nature. A total of 24 (21 for detection by microsatellites and three for detection by reisolation) 5-mm-long root segments, i.e. 12 cm of roots of the whole root system, were examined for the presence of PAC. This is a very short part of the more than 3 m mean total root length expected to be produced by Norway spruce seedlings under similar conditions as the ones applied in our experiment [13]. Thus, PAC probably colonized the roots of most seedlings but detection was not always successful. Nevertheless, a detection rate of 64% is still satisfactory.

Looking at fungal biomass, PAC colonization was denser in non-mycorrhized roots. This effect is supposed to be due to competition for space and nutrients or antagonism between *L. bicolor* and PAC. Again we have to take into account that *L. bicolor* was inoculated prior to PAC and was allowed to colonize the roots well in advance of PAC. It is well-known that timing of inoculation is influencing the competitive outcome of species interactions, favoring the leadoff species [1], [28], [49]. As Zak [37] suggested, ECM could protect the root against pathogens by utilizing surplus carbohydrates thus reducing attractiveness of the root to pathogens or by providing a physical barrier. Already Richard *et al.* [50] showed that the ectomycorrhizal fungus *Suillus granulatus* prevented DSE from adversely affecting *Picea mariana* seedlings, assuming that *S. granulatus* is preventing DSE from expressing its pathogenic effects. Protection against pathogens by ectomycorrhizae has been demonstrated for *Phytophthora cambivora* and *P. cinnamomi* causing ink disease on chestnut [51], *Cylindrocladium floridanum* causing root rot on conifers [52] or the root-pathogen *Fusarium oxysporum* on Douglas-fir [53]. One might object that PAC can also colonize roots undergoing secondary growth [17] whereas ectomycorrhizal fungi can colonize root tips only [28]. However, the root system of our plants was mostly primary since we worked with very young seedlings, and, thus, PAC and *L. bicolor* were ‘forced’ to compete because *L. bicolor* could be found in the whole root system, not only on the peripheral root-tips. Even though *L. bicolor* was inoculated earlier than PAC it was reduced by all four PAC strains in Norway spruce at 25°C though not significantly (Figure 4D).

Mycorrhized Norway spruce at 19°C inoculated with PAC strain D accumulated more biomass than plants assigned to any other treatment (Figure 2). A synergistic interaction between strain D, *L. bicolor* and their hosts might be a possible explanation for this effect. Perhaps, the interaction between *L. bicolor* and strain D triggered production of plant growth stimulating metabolites (hormones) [54]. On the other hand, this host-fungus interaction could have been inducing systemic resistance in the hosts and simultaneously triggered plant growth [55]–[57]. Strain D inoculated into mycorrhized roots could only be detected in Douglas-fir but not in Norway spruce either by microsatellites or reisolation. However, since the synergistic effect between strain D and *L. bicolor* is even stronger in Norway spruce we assume that some interactions between *L. bicolor* and strain D took place, possibly in the potting medium. Organic matter may have been decomposed by *L. bicolor* and/or strain D, and the released nutrients assimilated by the host plant promoting plant growth [14], [58].

Temperature had a considerable impact on biomass of Norway spruce, PAC biomass in Douglas-fir roots, root/shoot ratio and the degree of mycorrhization. The degree of mycorrhization was lower at higher temperature in both host plants (Figure 4) as also observed by Kasai *et al.* [59] on *Quercus myrsinaefolia*. When mycorrhization decreases other fungi including pathogens can occupy the freed niche, a process that probably rather harms than helps the host plant. Fungus-free control plants at 19°C accumulated more biomass than mycorrhized control plants. Even though not significant this indicates that *L. bicolor* also follows the mutualism-parasitism continuum described by Johnson *et al.* [7] whereupon ‘mycorrhizal fungi might be considered to be parasitic on plants when net cost of the symbiosis exceeds net benefits’.

Our results showed clearly that mycorrhization formed by *Laccaria bicolor* S238N reduced adverse effects of PAC on Norway spruce and Douglas-fir depending on the temperature. It is very likely that this mechanism functions in nature as well, since PAC and ECM live very closely together in natural habitats. This might be one part of the explanation why coniferous forests look healthy even though they are densely colonized by PAC and many other endophytes.

## Supporting Information

Table S1 **Factors in the full and reduced models with plant biomass as response variable.** The stepAIC command implemented in R was used to find the reduced models. Values are given for models including both hosts and with the two hosts separately. Significance level ≤0.05; ***, 0≤p≤0.001; **, 0.001<p≤0.01; *, 0.01<p≤0.05.
(PDF)

Table S2 **Factors in the full and reduced models with root/shoot biomass as response variable.** The stepAIC command implemented in R was used to find the reduced models. Values are given for models including both hosts and with the two hosts separately. Significance level ≤0.05; ***, 0≤p≤0.001; **, 0.001<p≤0.01; *, 0.01<p≤0.05.
(PDF)

Table S3 **Factors in the full and reduced models with fungal biomass as response variable.** The stepAIC command implemented in R was used to find the reduced models. Values are given for models with the two hosts combined as well as for each host separately. Significance level ≤0.05; ***, 0≤p≤0.001; **, 0.001<p≤0.01; *, 0.01<p≤0.05
(PDF)

Table S4 **Factors in the full and reduced models with mycorrhization as response variable.** The stepAIC command implemented in R was used to find the reduced models. Values are given for models with the two hosts combined as well as for each host separately. Significance level ≤0.05; ***, 0≤p≤0.001; **, 0.001<p≤0.01; *, 0.01<p≤0.05.
(PDF)
