# Supplementary material for: Mycorrhiza Reduces Adverse Effects of Dark Septate Endophytes (DSE) on Growth of Conifers
Source: PLoS One. 2012 Aug 10;7(8):e42865. doi: 10.1371/journal.pone.0042865 (PMC3416760; doi:10.1371/journal.pone.0042865)
Supplement: Table S1 — Factors in the full and reduced models with plant biomass as response variable. The stepAIC command implemented in R was used to find the reduced models. Values are given for models including both hosts and with the two hosts separately. Significance level ≤0.05; ***, 0≤p≤0.001; **, 0.001<p≤0.01; *, 0.01<p≤0.05. (PDF) [file pone.0042865.s001.pdf]

Table S1. Factors in the full and reduced models with plant biomass as response variable. The stepAIC command implemented in R was used to find the reduced models. Values are given for models including both hosts and with the two hosts separately. Significance level ≤ 0.05; ***, $0 \le p \le 0.001$; **, $0.001 < p \le 0.01$; *, $0.01 < p \le 0.05$

Both Hosts

Full Model

Factors	Df	Sum Sq	Mean Sq	F value	Pr(>F)	
PAC strain	3	0.12386	0.04129	7.2482	0.000127	***
Temperature	1	0.72964	0.72964	128.099	< 2.2e-16	***
Mycorrization	1	0.72782	0.72782	127.7801	< 2.2e-16	***
Host	1	1.61431	1.61431	283.4182	< 2.2e-16	***
Block	1	0.01729	0.01729	3.0358	0.0831173	.
PAC strain:Temperature	3	0.04487	0.01496	2.6256	0.0518466	.
PAC strain:Mycorrization	3	0.0421	0.01403	2.4637	0.0638824	.
Temperature:Mycorrization	1	0.00186	0.00186	0.3259	0.568795	
PAC strain:Host	3	0.03875	0.01292	2.2677	0.0821568	.
Temperature:Host	1	0.8131	0.8131	142.7517	< 2.2e-16	***
Mycorrization:Host	1	0.15068	0.15068	26.4551	6.87E-07	***
PAC strain:Block	3	0.0065	0.00217	0.3805	0.767155	
Temperature:Block	1	0.00138	0.00138	0.2427	0.6228609	
Mycorrization:Block	1	0.00001	0.00001	0.0013	0.9709123	
Host:Block	1	0.00005	0.00005	0.0096	9.22E-01	
PAC strain:Temperature:Mycorrization	3	0.0489	0.0163	2.8619	0.0381878	*
PAC strain:Temperature:Host	3	0.03429	0.01143	2.0069	0.1145541	
PAC strain:Mycorrization:Host	3	0.03547	0.01182	2.0755	0.1049871	
Temperature:Mycorrization:Host	1	0.09942	0.09942	17.4553	4.54E-05	***
PAC strain:Temperature:Block	3	0.02251	0.0075	1.3172	0.2702092	
PAC strain:Mycorrization:Block	3	0.0158	0.00527	0.9247	0.429994	
Temperature:Mycorrization:Block	1	0.01454	0.01454	2.5527	0.1118189	
PAC strain:Host:Block	3	0.00562	0.00187	0.3289	0.8044622	
Temperature:Host:Block	1	0.02223	0.02223	3.9029	0.0496988	*
Mycorrization:Host:Block	1	0.00191	0.00191	0.3347	5.64E-01	
PAC strain:Temperature:Mycorrization:Host	3	0.06314	0.02105	3.695	0.0128992	*
PAC strain:Temperature:Mycorrization:Block	3	0.02819	0.0094	1.6498	0.1794719	
PAC strain:Temperature:Host:Block	3	0.00179	0.0006	0.1045	0.9573613	
PAC strain:Mycorrization:Host:Block	3	0.01307	0.00436	0.7646	0.5152255	
Temperature:Mycorrization:Host:Block	1	0.00238	0.00238	0.4175	0.5190053	
PAC strain:Temperature:Mycorrization:Host:Block	3	0.00289	0.00096	0.1689	0.9172679	

Reduced Model	**Factors**	**Df**	**Sum Sq**	**Mean Sq**	**F value**	**Pr(>F)**	
	PAC strain	3	0.12386	0.04129	7.4982	8.96E-03	***
	Temperature	1	0.72964	0.72964	132.5175	< 2.2e-16	***
	Mycorrization	1	0.72782	0.72782	132.1875	< 2.2e-16	***
	Host	1	1.61431	1.61431	293.194	< 2.2e-16	***
	Block	1	0.01729	0.01729	3.1405	0.07794	.
	PAC strain:Temperature	3	0.04487	0.01496	2.7162	0.04596	*
	PAC strain:Mycorrization	3	0.0421	0.01403	2.5487	0.05709	.
	Temperature:Mycorrization	1	0.00186	0.00186	0.3371	0.56217	
	PAC strain:Host	3	0.03875	0.01292	2.3459	0.07413	.
	Temperature:Host	1	0.8131	0.8131	147.6756	< 2.2e-16	***
	Mycorrization:Host	1	0.15068	0.15068	27.3676	4.34E-05	***
	PAC strain:Block	3	0.0065	0.00217	0.3937	0.75771	
	Temperature:Block	1	0.00138	0.00138	0.2511	0.6169	
	Mycorrization:Block	1	0.00001	0.00001	0.0014	0.97041	
	Host:Block	1	0.00005	0.00005	0.0099	0.92077	
	PAC strain:Temperature:Mycorrization	3	0.0489	0.0163	2.9606	0.03346	*
	PAC strain:Temperature:Host	3	0.03429	0.01143	2.0761	0.10471	
	PAC strain:Mycorrization:Host	3	0.03547	0.01182	2.1471	0.09564	.
	Temperature:Mycorrization:Host	1	0.09942	0.09942	18.0574	3.33E-03	***
	PAC strain:Temperature:Block	3	0.02251	0.0075	1.3626	0.25551	
	PAC strain:Mycorrization:Block	3	0.0158	0.00527	0.9566	0.41436	
	Temperature:Mycorrization:Block	1	0.01454	0.01454	2.6408	0.10578	
	PAC strain:Host:Block	3	0.00562	0.00187	0.3402	0.79625	
	Temperature:Host:Block	1	0.02223	0.02223	4.0375	0.04589	*
	Mycorrization:Host:Block	1	0.00191	0.00191	0.3463	0.55692	
	PAC strain:Temperature:Mycorrization:Host	3	0.06314	0.02105	3.8225	0.01084	*
	PAC strain:Temperature:Mycorrization:Block	3	0.02819	0.0094	1.7067	0.16697	
	Residuals	194	1.06816	0.00551			

Douglas-Fir

Full Model	**Factors**	**Df**	**Sum Sq**	**Mean Sq**	**F value**	**Pr(>F)**	
	Temperature	1	0.0013	0.001304	0.1563	0.693573	
	PAC strain	3	0.03983	0.013277	1.5907	0.197344	
	Mycorrhization	1	0.21128	0.21128	25.3129	2.56E-06	***
	Block	1	0.01463	0.01463	1.7528	0.188957	
	Temperature:PAC strain	3	0.03225	0.010749	1.2878	0.283618	
	Temperature:Mycorrhization	1	0.10229	0.102285	12.2545	0.000731	***
	PAC strain:Mycorrhization	3	0.00463	0.001543	0.1848	0.906449	
	Temperature:Block	1	0.01885	0.01885	2.2583	0.136477	
	PAC strain:Block	3	0.00192	0.000639	0.0765	0.972497	
	Mycorrhization:Block	1	0.00159	0.001588	0.1903	0.663758	
	Temperature:PAC strain:Mycorrhization	3	0.01085	0.003617	0.4333	0.729667	
	Temperature:PAC strain:Block	3	0.01929	0.006431	0.7705	0.513557	
	Temperature:Mycorrhization:Block	1	0.0046	0.004601	0.5512	0.459798	
	PAC strain:Mycorrhization:Block	3	0.03346	0.011152	1.3361	0.267805	
	Temperature:PAC strain:Mycorrhization:Block	3	0.03652	0.012175	1.4586	0.231357	
	Residuals	88	0.73451	0.008347			

Reduced Model	**Factors**	**Df**	**Sum Sq**	**Mean Sq**	**F value**	**Pr(>F)**	
	Temperature	1	0.0013	0.001304	0.163	0.6871897	
	Mycorrhization	1	0.21536	0.215359	26.9089	9.37E-07	***
	Block	1	0.01235	0.012354	1.5436	0.2166296	
	Temperature:Mycorrhization	1	0.10695	0.10695	13.3633	0.0003896	***
	Temperature:Block	1	0.01946	0.019461	2.4316	0.1216809	
	Residuals	114	0.91237	0.008003			

Picea

Full Model

Factors	Df	Sum Sq	Mean Sq	F value	Pr(>F)	
Temperature	1	2.02066	2.02066	249.1859	< 2.2e-16	***
PAC strain	3	0.19898	0.06633	8.1795	6.64E-05	***
Mycorrhization	1	0.84077	0.84077	103.683	< 2.2e-16	***
Block	1	0.02822	0.02822	3.48	0.065169	.
Temperature:PAC strain	3	0.06369	0.02123	2.618	0.055339	.
Temperature:Mycorrhization	1	0.00878	0.00878	1.0833	0.300568	
PAC strain:Mycorrhization	3	0.13295	0.04432	5.4652	0.001642	**
Temperature:Block	1	0.01086	0.01086	1.3391	0.250073	
PAC strain:Block	3	0.01864	0.00621	0.7664	0.515619	
Mycorrhization:Block	1	0.00011	0.00011	0.0134	0.908077	
Temperature:PAC strain:Mycorrhization	3	0.13205	0.04402	5.428	0.001718	**
Temperature:PAC strain:Block	3	0.01128	0.00376	0.4638	0.708197	
Temperature:Mycorrhization:Block	1	0.00946	0.00946	1.1671	0.282712	
PAC strain:Mycorrhization:Block	3	0.00949	0.00316	0.3899	0.760525	
Temperature:PAC strain:Mycorrhization:Block	3	0.02375	0.00792	0.9762	0.407351	
Residuals	96	0.77847	0.00811			

Reduced Model

Factors	Df	Sum Sq	Mean Sq	F value	Pr(>F)	
Temperature	1	2.02066	2.02066	259.2496	< 2.2e-16	***
PAC strain	3	0.19898	0.06633	8.5098	3.89E-05	***
Mycorrhization	1	0.84077	0.84077	107.8704	< 2.2e-16	***
Block	1	0.02822	0.02822	3.6205	0.059662	.
Temperature:Mycorrhization	1	0.00878	0.00878	1.1271	0.290703	
Temperature:PAC strain	3	0.06369	0.02123	2.7237	0.047705	*
PAC strain:Mycorrhization	3	0.13295	0.04432	5.686	0.001168	**
Temperature:PAC strain:Mycorrhization	3	0.12894	0.04298	5.5145	0.001443	**
Residuals	111	0.86516	0.00779			
