# Supplementary material for: Mycorrhiza Reduces Adverse Effects of Dark Septate Endophytes (DSE) on Growth of Conifers
Source: PLoS One. 2012 Aug 10;7(8):e42865. doi: 10.1371/journal.pone.0042865 (PMC3416760; doi:10.1371/journal.pone.0042865)
Supplement: Table S2 — Factors in the full and reduced models with root/shoot biomass as response variable. The stepAIC command implemented in R was used to find the reduced models. Values are given for models including both hosts and with the two hosts separately. Significance level ≤0.05; ***, 0≤p≤0.001; **, 0.001<p≤0.01; *, 0.01<p≤0.05. (PDF) [file pone.0042865.s002.pdf]

Table S2. Factors in the full and reduced models with root/shoot biomass as response variable. The stepAIC command implemented in R was used to find the reduced models. Values are given for models including both hosts and with the two hosts separately. Significance level ≤ 0.05; ***, 0 ≤ p ≤ 0.001; **, 0.001 < p ≤ 0.01; *, 0.01 < p ≤ 0.05

Both Hosts

Full Model

Factors	Df	Sum Sq	Mean Sq	F value	Pr(>F)	
PAC strain	3	1.493	0.4977	1.5338	0.20724	
Temperature	1	7.91	7.9104	24.3806	1.766e-06	***
Mycorrhization	1	0.459	0.4595	1.4161	0.23558	
Host	1	0.056	0.0561	0.1729	0.67806	
Block	1	0.83	0.8297	2.5571	0.11152	
PAC strain:Temperature	3	1.361	0.4535	1.3977	0.24497	
PAC strain:Mycorrhization	3	0.805	0.2684	0.8271	0.48047	
Temperature:Mycorrhization	1	0.258	0.2578	0.7944	0.37393	
PAC strain:Host	3	0.609	0.2029	0.6255	0.59941	
Temperature:Host	1	0.04	0.0404	0.1245	0.72464	
Mycorrhization:Host	1	1.761	1.7611	5.4279	0.0209	*
PAC strain:Block	3	1.33	0.4433	1.3663	0.25455	
Temperature:Block	1	0.027	0.0267	0.0822	0.7746	
Mycorrhization:Block	1	0.315	0.315	0.9707	0.32579	
Host:Block	1	0.069	0.0693	0.2134	0.64462	
PAC strain:Temperature:Mycorrhization	3	0.792	0.2641	0.8139	0.48769	
PAC strain:Temperature:Host	3	1.342	0.4472	1.3782	0.25087	
PAC strain:Mycorrhization:Host	3	0.318	0.1059	0.3265	0.8062	
Temperature:Mycorrhization:Host	1	0.74	0.74	2.2807	0.1327	
PAC strain:Temperature:Block	3	0.475	0.1582	0.4877	0.69123	
PAC strain:Mycorrhization:Block	3	0.388	0.1293	0.3985	0.75423	
Temperature:Mycorrhization:Block	1	0.008	0.0076	0.0233	0.87892	
PAC strain:Host:Block	3	0.382	0.1275	0.3929	0.75825	
Temperature:Host:Block	1	0.368	0.3677	1.1332	0.28849	
Mycorrhization:Host:Block	1	0.001	0.0006	0.0019	0.96499	
PAC strain:Temperature:Mycorrhization:Host	3	0.753	0.251	0.7735	0.51019	
PAC strain:Temperature:Mycorrhization:Block	3	0.31	0.1033	0.3183	0.81215	
PAC strain:Temperature:Host:Block	3	1.209	0.403	1.2421	0.29585	
PAC strain:Mycorrhization:Host:Block	3	1.063	0.3544	1.0923	0.35369	
Temperature:Mycorrhization:Host:Block	1	0.018	0.0182	0.0562	0.81283	
PAC strain:Temperature:Mycorrhization:Host:Block	3	0.298	0.0995	0.3066	0.82059	
Residuals	184	59.7	0.3245			

Reduced Model

Factors	Df	Sum Sq	Mean Sq	F value	Pr(>F)	
Temperature	1	7.953	7.9528	25.9325	7.17E-07	***
Mycorrhization	1	0.47	0.4697	1.5316	2.17E-01	
Host	1	0.06	0.0598	0.195	0.65919	
Block	1	0.898	0.8979	2.928	0.08835	.
Temperature:Mycorrhization	1	0.25	0.2498	0.8146	0.36767	
Temperature:Host	1	0.035	0.0347	0.1131	0.73691	
Mycorrhization:Host	1	1.718	1.7184	5.6034	0.01872	*
Temperature:Mycorrhization:Host	1	0.81	0.8096	2.64	0.10552	
Residuals	239	73.294	0.3067			

Douglas-fir

Full Model

Factors	Df	Sum Sq	Mean Sq	F value	Pr(>F)	
PAC strain	3	0.6409	0.21362	2.3328	0.07954	.
Mycorrhization	1	0.3877	0.38774	4.2342	0.04258	*
Temperature	1	3.0807	3.08075	33.6425	1.02E-07	***
Block	1	0.1647	0.16472	1.7988	0.18331	
PAC strain:Mycorrhization	3	0.0576	0.01918	0.2095	0.88958	
PAC strain:Temperature	3	0.375	0.125	1.3651	0.25874	
Mycorrhization:Temperature	1	0.0484	0.04837	0.5282	0.4693	
PAC strain:Block	3	0.2405	0.08018	0.8755	0.457	
Mycorrhization:Block	1	0.1556	0.15559	1.6991	0.19581	
Temperature:Block	1	0.2758	0.27579	3.0117	0.08617	.
PAC strain:Mycorrhization:Temperature	3	0.1771	0.05903	0.6446	0.58839	
PAC strain:Mycorrhization:Block	3	0.2319	0.07731	0.8442	0.47331	
PAC strain:Temperatureerature:Block	3	0.1672	0.05572	0.6085	0.61122	
Mycorrhization:Temperature:Block	1	0	0.00003	0.0003	0.98533	
PAC strain:Mycorrhization:Temperature:Block	3	0.1765	0.05884	0.6426	0.58967	
Residuals	88	8.0584	0.09157			

Reduced Model

Factors	Df	Sum Sq	Mean Sq	F value	Pr(>F)	
PAC strain	3	0.6409	0.21362	2.4766	0.06505	.
Mycorrhization	1	0.3877	0.38774	4.4952	0.0362	*
Temperature	1	3.0807	3.08075	35.7166	2.76E-08	***
Block	1	0.1647	0.16472	1.9097	0.16975	
Temperature:Block	1	0.3034	0.30343	3.5178	0.06332	.
Residuals	112	9.6606	0.08626			

Picea

Full Model

Factors	Df	Sum Sq	Mean Sq	F value	Pr(>F)	
Temperature	1	4.739	4.7395	8.8106	0.003781	**
PAC strain	3	1.355	0.4516	0.8396	0.475441	
Mycorrhization	1	1.992	1.992	3.703	0.057277	.
Block	1	0.683	0.683	1.2697	0.262625	
Temperature:PAC strain	3	2.444	0.8147	1.5146	0.215663	
Temperature:Mycorrhization	1	1.057	1.0567	1.9644	0.16427	
PAC strain:Mycorrhization	3	1.042	0.3474	0.6458	0.587458	
Temperature:Block	1	0.133	0.1329	0.247	0.620331	
PAC strain:Block	3	1.268	0.4227	0.7858	0.504698	
Mycorrhization:Block	1	0.217	0.2171	0.4037	0.52671	
Temperature:PAC strain:Mycorrhization	3	1.538	0.5127	0.9531	0.418255	
Temperature:PAC strain:Block	3	1.504	0.5014	0.9321	0.428377	
Temperature:Mycorrhization:Block	1	0.041	0.0414	0.0769	0.782179	
PAC strain:Mycorrhization:Block	3	1.038	0.3461	0.6434	0.588948	
Temperature:PAC strain:Mycorrhization:Block	3	0.539	0.1796	0.3338	0.800928	
Residuals	96	51.642	0.5379			

Reduced Model

Factors	Df	Sum Sq	Mean Sq	F value	Pr(>F)	
Temperature	1	4.739	4.7395	9.2559	0.002866	**
Mycorrhization	1	1.992	1.992	3.8902	0.050795	.
Temperature:Mycorrhization	1	1.007	1.0073	1.9672	0.163241	
Residuals	124	63.494	0.5121			
