# Supplementary material for: Mycorrhiza Reduces Adverse Effects of Dark Septate Endophytes (DSE) on Growth of Conifers
Source: PLoS One. 2012 Aug 10;7(8):e42865. doi: 10.1371/journal.pone.0042865 (PMC3416760; doi:10.1371/journal.pone.0042865)
Supplement: Table S3 — Factors in the full and reduced models with fungal biomass as response variable. The stepAIC command implemented in R was used to find the reduced models. Values are given for models with the two hosts combined as well as for each host separately. Significance level ≤0.05; ***, 0≤p≤0.001; **, 0.001<p≤0.01; *, 0.01<p≤0.05 (PDF) [file pone.0042865.s003.pdf]

Table S3. Factors in the full and reduced models with fungal biomass as response variable. The stepAIC command implemented in R was used to find the reduced models. Values are given for models with the two hosts combined as well as for each host separately. Significance level ≤ 0.05; ***, 0 ≤ p ≤ 0.001; **, 0.001 < p ≤ 0.01; *, 0.01 < p ≤ 0.05

Both Hosts

Full Model

Factors	Df	Sum Sq	Mean Sq	F value	Pr(>F)	
PAC strain	4	0.0012054	0.0003014	14.5345	1.38E-07	***
Temperature	1	0.0001496	0.0001496	7.2161	0.0102279	*
Mycorrhization	1	0.0007649	0.0007649	36.8897	2.86E-07	***
Host	1	0.0009024	0.0009024	43.5251	4.94E-08	***
Block	1	0.0002906	0.0002906	14.014	0.0005339	***
PAC strain:Temperature	4	0.0001002	2.505E-05	1.2081	0.3212878	
PAC strain:Mycorrhization	4	0.0002317	5.792E-05	2.7936	0.0378574	*
Temperature:Mycorrhization	1	0.0001457	0.0001457	7.0262	0.011198	*
PAC strain:Host	4	0.0004006	0.0001002	4.8303	0.002627	**
Temperature:Host	1	0.0002606	0.0002606	12.5703	0.0009603	***
Mycorrhization:Host	1	0.0001221	0.0001221	5.8888	0.0195025	*
PAC strain:Block	4	5.728E-05	1.432E-05	0.6907	0.602417	
Temperature:Block	1	2.304E-05	2.304E-05	1.1112	0.2977064	
Mycorrhization:Block	1	4.699E-05	4.699E-05	2.2666	0.1395027	
Host:Block	1	5.028E-05	5.028E-05	2.4252	0.1267253	
PAC strain:Temperature:Mycorrhization	4	0.0001105	2.762E-05	1.3322	0.2734162	
PAC strain:Temperature:Host	4	0.0002395	5.988E-05	2.8879	0.0333211	*
PAC strain:Mycorrhization:Host	4	8.778E-05	2.195E-05	1.0585	0.3886643	
Temperature:Mycorrhization:Host	1	0.0001548	0.0001548	7.4665	0.0090842	**
PAC strain:Temperature:Block	4	0.0000748	0.0000187	0.9019	0.4713059	
PAC strain:Mycorrhization:Block	4	4.431E-05	1.108E-05	0.5343	0.7112155	
Temperature:Mycorrhization:Block	1	1.804E-05	1.804E-05	0.8701	0.3561246	
PAC strain:Host:Block	4	2.284E-05	5.71E-06	0.2754	0.8922574	
Temperature:Host:Block	1	7E-08	7E-08	0.0036	0.952523	
Mycorrhization:Host:Block	1	1.241E-05	1.241E-05	0.5984	0.4434263	
PAC strain:Temperature:Mycorrhization:Host	4	0.0002024	5.059E-05	2.4401	0.0612299	.
PAC strain:Temperature:Mycorrhization:Block	4	3.881E-05	0.0000097	0.468	0.7588823	
PAC strain:Temperature:Host:Block	4	5.26E-06	1.32E-06	0.0634	0.9923168	
PAC strain:Mycorrhization:Host:Block	3	0.0002457	8.191E-05	3.9506	0.0141904	*
Temperature:Mycorrhization:Host:Block	1	1.012E-05	1.012E-05	0.4882	0.4884979	
PAC strain:Temperature:Mycorrhization:Host:Block	2	7.471E-05	3.735E-05	1.8017	0.1772578	
Residuals	43	0.0008915	2.073E-05			

Reduced Model This model correponds to the full model for both hosts; no reduction possible using the stepAIC command implemented in the statistic package 'R'

Douglas-Fir

Full Model

Factors	Df	Sum Sq	Mean Sq	F value	Pr(>F)	
PAC strain	4	0.0013843	0.0003461	10.5863	6.15E-05	***
Temperature	1	0.0004275	0.0004275	13.0769	0.001531	**
Mycorrhization	1	0.0008442	0.0008442	25.8227	4.33E-05	***
Block	1	0.0002901	0.0002901	8.8731	0.006927	**
PAC strain:Temperature	4	0.0003246	8.114E-05	2.4819	0.073501	.
PAC strain:Mycorrhization	4	0.0002266	5.664E-05	1.7325	0.17877	
Temperature:Mycorrhization	1	0.0002949	0.0002949	9.0211	0.006541	**
PAC strain:Block	4	5.608E-05	1.402E-05	0.4288	0.78623	
Temperature:Block	1	9.83E-06	9.83E-06	0.3007	0.588984	
Mycorrhization:Block	1	0.0000011	0.0000011	0.0337	0.856044	
PAC strain:Temperature:Mycorrhization	4	0.0002762	6.906E-05	2.1123	0.1135	
PAC strain:Temperature:Block	4	5.027E-05	1.257E-05	0.3844	0.817397	
PAC strain:Mycorrhization:Block	3	0.0002428	8.094E-05	2.4759	0.088137	.
Temperature:Mycorrhization:Block	1	1.55E-06	1.55E-06	0.0474	0.829655	
PAC strain:Temperature:Mycorrhization:Block	3	6.158E-05	2.053E-05	0.6279	0.604681	
Residuals	22	0.0007192	3.269E-05			

Reduced Model	Factors	Df	Sum Sq	Mean Sq	F value	Pr(>F)	
	PAC strain	4	0.0013843	0.0003461	12.4698	4.33E-06	***
	Temperature	1	0.0004275	0.0004275	15.4035	0.0004694	***
	Mycorrhization	1	0.0008442	0.0008442	30.417	5.44E-06	***
	Block	1	0.0002901	0.0002901	10.4518	0.0029747	**
	PAC strain:Temperature	4	0.0003246	8.114E-05	2.9234	0.0373547	*
	PAC strain:Mycorrhization	4	0.0002266	5.664E-05	2.0408	0.1138363	
	Temperature:Mycorrhization	1	0.0002949	0.0002949	10.6261	0.0027749	**
	PAC strain:Block	4	5.608E-05	1.402E-05	0.5051	0.732244	
	Temperature:Block	1	9.83E-06	9.83E-06	0.3542	0.5562277	
	Mycorrhization:Block	1	0.0000011	0.0000011	0.0397	0.8434388	
	PAC strain:Temperature:Mycorrhization	4	0.0002762	6.906E-05	2.4882	0.0644322	.
	PAC strain:Mycorrhization:Block	3	0.0002428	8.094E-05	2.9165	0.0503087	.
	Residuals	30	0.0008326	2.775E-05			

Picea

Full Model	Factors	Df	Sum Sq	Mean Sq	F value	Pr(>F)	
	PAC strain	4	1.71E-04	4.27E-05	5.2032	0.004506	**
	Temperature	1	1.14E-05	1.14E-05	1.3906	0.251486	
	Mycorrhization	1	1.01E-04	1.01E-04	12.3248	0.002079	**
	Block	1	3.15E-05	3.15E-05	3.8395	0.063467	.
	PAC strain:Temperature	4	2.72E-05	6.79E-06	0.8274	0.52259	
	PAC strain:Mycorrhization	4	9.21E-05	2.30E-05	2.8072	0.051878	.
	Temperature:Mycorrhization	1	0.00E+00	0.00E+00	0.0001	0.994179	
	PAC strain:Block	4	2.41E-05	6.04E-06	0.7357	0.577958	
	Temperature:Block	1	1.46E-05	1.46E-05	1.7827	0.196116	
	Mycorrhization:Block	1	2.87E-05	2.87E-05	3.4917	0.075692	.
	PAC strain:Temperature:Mycorrhization	4	5.08E-05	1.27E-05	1.5473	0.225081	
	PAC strain:Temperature:Block	4	2.56E-05	6.39E-06	0.7787	0.551505	
	PAC strain:Mycorrhization:Block	4	6.63E-05	1.66E-05	2.0185	0.12863	
	Temperature:Mycorrhization:Block	1	2.82E-05	2.82E-05	3.4345	0.077953	.
	PAC strain:Temperature:Mycorrhization:Block	3	2.70E-05	9.01E-06	1.0975	0.372154	
	Residuals	21	1.72E-04	8.21E-06			

Reduced Model	Factors	Df	Sum Sq	Mean Sq	F value	Pr(>F)	
	PAC strain	4	1.71E-04	4.27E-05	5.2032	0.004506	**
	Temperature	1	1.14E-05	1.14E-05	1.3906	0.251486	
	Mycorrhization	1	1.01E-04	1.01E-04	12.3248	0.002079	**
	Block	1	3.15E-05	3.15E-05	3.8395	0.063467	.
	PAC strain:Temperature	4	2.72E-05	6.79E-06	0.8274	0.52259	
	PAC strain:Mycorrhization	4	9.21E-05	2.30E-05	2.8072	0.051878	.
	Temperature:Mycorrhization	1	0.00E+00	0.00E+00	0.0001	0.994179	
	PAC strain:Block	4	2.41E-05	6.04E-06	0.7357	0.577958	
	Temperature:Block	1	1.46E-05	1.46E-05	1.7827	0.196116	
	Mycorrhization:Block	1	2.87E-05	2.87E-05	3.4917	0.075692	.
	PAC strain:Temperature:Mycorrhization	4	5.08E-05	1.27E-05	1.5473	0.225081	
	PAC strain:Temperature:Block	4	2.56E-05	6.39E-06	0.7787	0.551505	
	PAC strain:Mycorrhization:Block	4	6.63E-05	1.66E-05	2.0185	0.12863	
	Temperature:Mycorrhization:Block	1	2.82E-05	2.82E-05	3.4345	0.077953	.
	PAC strain:Temperature]:Mycorrhization:Block	3	2.70E-05	9.01E-06	1.0975	0.372154	
	Residuals	21	1.72E-04	8.21E-06			
