# Supplementary material for: Mycorrhiza Reduces Adverse Effects of Dark Septate Endophytes (DSE) on Growth of Conifers
Source: PLoS One. 2012 Aug 10;7(8):e42865. doi: 10.1371/journal.pone.0042865 (PMC3416760; doi:10.1371/journal.pone.0042865)
Supplement: Table S4 — Factors in the full and reduced models with mycorrhization as response variable. The stepAIC command implemented in R was used to find the reduced models. Values are given for models with the two hosts combined as well as for each host separately. Significance level ≤0.05; ***, 0≤p≤0.001; **, 0.001<p≤0.01; *, 0.01<p≤0.05. (PDF) [file pone.0042865.s004.pdf]

Table S4. Factors in the full and reduced models with mycorrhization as response variable. The stepAIC command implemented in R was used to find the reduced models. Values are given for models with the two hosts combined as well as for each host separately. Significance level ≤ 0.05; ***, 0 ≤ p ≤ 0.001; **, 0.001 < p ≤ 0.01; *, 0.01 < p ≤ 0.05

Both Hosts

Full Model

Factors	Df	Sum Sq	Mean Sq	F value	Pr(>F)	
PAC strain	4	0.71	0.1771	0.1471	0.9641855	
Temperature	1	31.15	31.1529	25.8725	6.82E-07	***
Block	1	16.71	16.7115	13.8789	0.0002373	***
Host	1	0.23	0.2321	0.1928	0.6609801	
PAC strain:Temperature	4	1.13	0.2813	0.2336	0.9192941	
PAC strain:Block	4	0.58	0.1458	0.1211	0.974871	
Temperature:Block	1	19.09	19.0882	15.8528	8.80E-05	***
PAC strain:Host	4	1.35	0.3378	0.2806	0.890443	
Temperature:Host	1	0.05	0.0508	0.0422	0.8373804	
Block:Host	1	1.09	1.0915	0.9065	0.3419002	
PAC strain:Temperature:Block	4	0.06	0.0151	0.0125	0.9996896	
PAC strain:Temperature:Host	4	0.76	0.1907	0.1583	0.9591014	
PAC strain:Block:Host	4	1.36	0.3409	0.2831	0.8888097	
Temperature:Block:Host	1	0.55	0.5511	0.4577	0.4992826	
Residuals	271	326.31	1.2041			

Reduced Model

Factors	Df	Sum Sq	Mean Sq	F value	Pr(>F)	
Temperature	1	3.12E+01	31.1834	28.444	1.88E-07	***
Block	1	16.43	16.4318	14.988	0.0001322	***
Temperature:Block	1	1.84E+01	18.4401	16.82	5.27E-05	***
Residuals	156	1.49E+02	0.9538			

Douglas-Fir

Full Model

Factors	Df	Sum Sq	Mean Sq	F value	Pr(>F)	
PAC strain	4	1.82	0.4549	0.3272	0.85931	
Temperature	1	15.454	15.4541	11.1146	0.001114	**
Block	1	3.941	3.9406	2.8341	0.094665	.
PAC strain:Temperature	4	1.023	0.2558	0.184	0.946375	
PAC strain:Block	4	1.306	0.3266	0.2349	0.918283	
Temperature:Block	1	5.892	5.8918	4.2374	0.041525	*
PAC strain:Temperature:Block	4	0.603	0.1508	0.1085	0.979383	
Residuals	131	182.146	1.3904			

Reduced Model

Factors	Df	Sum Sq	Mean Sq	F value	Pr(>F)	
Temperature	1	15.678	15.6782	12.065	0.0006742	***
Block	1	4.177	4.1767	3.214	0.0750523	.
Residuals	148	192.331	1.2995			

Picea

Full Model

Factors	Df	Sum Sq	Mean Sq	F value	Pr(>F)	
PAC strain	4	1.063	0.2656	0.258	0.9043894	
Temperature	1	15.625	15.625	15.1738	0.0001513	***
Block	1	13.715	13.7155	13.3195	0.0003699	***
PAC strain:Temperature	4	1.262	0.3155	0.3064	0.8733209	
PAC strain:Block	4	0.74	0.1849	0.1796	0.9486547	
Temperature:Block	1	12.886	12.8865	12.5144	0.0005483	***
PAC strain:Temperature:Block	4	0.921	0.2303	0.2237	0.9248217	
Residuals						

Reduced Model

Factors	Df	Sum Sq	Mean Sq	F value	Pr(>F)	
Temperature	1	15.625	15.625	16.382	8.13E-05	***
Block	1	13.535	13.5352	14.191	0.0002337	***
Temperature:Block	1	12.42	12.4203	13.022	0.0004142	***
Residuals	156	148.795	0.9538			
